# Fractionation of a Herbal Antidiarrheal Medicine Reveals Eugenol as an Inhibitor of Ca^2+^-Activated Cl^−^ Channel TMEM16A

**DOI:** 10.1371/journal.pone.0038030

**Published:** 2012-05-30

**Authors:** Zhen Yao, Wan Namkung, Eun A. Ko, Jinhong Park, Lukmanee Tradtrantip, A. S. Verkman

**Affiliations:** 1 Departments of Medicine and Physiology, University of California San Francisco, San Francisco, California, United States of America; 2 Yonsei University, College of Pharmacy, Yonsei Institute of Pharmaceutical Sciences, Incheon, Korea; Dalhousie University, Canada

## Abstract

The Ca^2+^-activated Cl^−^ channel TMEM16A is involved in epithelial fluid secretion, smooth muscle contraction and neurosensory signaling. We identified a Thai herbal antidiarrheal formulation that inhibited TMEM16A Cl^−^ conductance. C18-reversed-phase HPLC fractionation of the herbal formulation revealed >98% of TMEM16A inhibition activity in one out of approximately 20 distinct peaks. The purified, active compound was identified as eugenol (4-allyl-2-methoxyphenol), the major component of clove oil. Eugenol fully inhibited TMEM16A Cl^−^ conductance with single-site IC_50_∼150 µM. Eugenol inhibition of TMEM16A in interstitial cells of Cajal produced strong inhibition of intestinal contraction in mouse ileal segments. TMEM16A Cl^−^ channel inhibition adds to the list of eugenol molecular targets and may account for some of its biological activities.

## Introduction

Intestinal fluid secretion in secretory diarrheas involves Cl^−^ movement from the blood to the intestinal lumen through Cl^−^ channels on the enterocyte apical plasma membrane. These Cl^−^ channels include a cAMP-gated channel, CFTR (cystic fibrosis transmembrane conductance regulator), and Ca^2+^-activated Cl^−^ channels (CaCCs), one of which has been identified as TMEM16A [1], [2]. While CFTR probably provides the primary route for Cl^−^ transport in enterotoxin-mediated secretory diarrheas such as cholera, CaCCs are likely involved as well and may provide the primary route for Cl^−^ transport in some viral, drug-induced and AIDS-related diarrheas [3]–[5].

TMEM16A (alternative name, ANO1) has been identified as a CaCC that is broadly expressed in tracheal, intestinal and glandular epithelia, smooth muscle cells, and gastrointestinal interstitial cells of Cajal, where it is involved in epithelial fluid secretion, smooth muscle contraction and gastrointestinal motility [6]–[8]. TMEM16A is also expressed in various tumors, where it may play a role in tumor cell proliferation [6]. We previously identified, by high-throughput screening, several small-molecule inhibitors and activators of TMEM16A Cl^−^ conductance [9], [10], which have potential therapeutic value in cystic fibrosis, dry mouth, gastric hypomotility (activators), secretory diarrhea, pain and tumor growth (inhibitors). We also discovered that gallotannin-containing red wines and green teas inhibit CaCC/TMEM16A activity, which may account for their reported beneficial effects in cardiovascular disease and secretory diarrheas [11].

Here, we identified an antidiarrheal herbal medicinal formulation with TMEM16A inhibition activity, which, upon purification and characterization, was attributed to eugenol, a major component of clove oil [12]. Despite its small molecular size, eugenol has been reported to have a wide range of biological activities. Eugenol is an oxygen radical scavenger and can prevent chemically induced organ damage by reducing lipid peroxidation [13]–[15]. Eugenol slows the growth of some tumors by reducing cell proliferation and increasing apoptosis [16], [17]. Eugenol also inhibits cyclooxygenase, inhibiting the biosynthesis of prostanoids, which cause pain, inflammation and carcinogenesis [18]. Here, we report a new activity for eugenol – Cl^−^ channel inhibition – that may account for some of its biological activities including analgesia and tumor suppression.

## Results

### TMEM16A inhibition by a Thai herbal formulation

**Figure 1:**
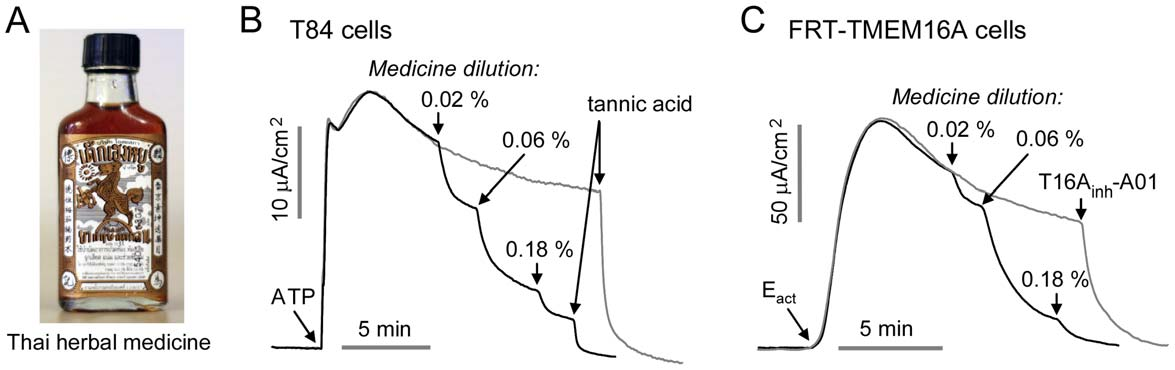
Inhibition of Ca^2+^-activated Cl^−^ channels by a Thai herbal formulation. A. Thai herbal medicine for diarrhea. B. Short-circuit measurement in T84 cells. Representative current trace (dark curve) shows inhibition of ATP (100 µM)-stimulated native CaCC Cl^−^ current by the Thai herbal formulation. Current in the control study (gray curve) without the herbal formulation was inhibited with 100 µM tannic acid. C. Current measurement in TMEM16A-transfected FRT cells shows inhibition of E_act_ (10 µM, a TMEM16A activator)-stimulated TMEM16A Cl^−^ current by the Thai herbal formulation (dark curve). Control study (gray curve) without the herbal formulation shows that 10 µM T16A_inh_-A01 (TMEM16A-selective inhibitor) totally inhibited the current.

Testing of eight Asian antidiarrheal remedies revealed inhibition of intestinal CaCC/TMEM16A by a Thai herbal formulation (Fig. 1A), which consists of a dark brown, pungent liquid containing a small amount of precipitate. Short-circuit current measurement in Fig. 1B shows inhibition of ATP-stimulated CaCC activity in T84 human colonic epithelial cells, with 50% inhibition at ∼0.05% (1∶2000 dilution) of the original formulation. The CaCC inhibitor tannic acid completely inhibited Cl^−^ current. Fig. 1C shows inhibition of TMEM16A Cl^−^ current in E_act_-simulated FRT cells expressing human TMEM16A. E_act_ is a TMEM16A-selective activator [10]. The TMEM16A-selective inhibitor T16A_inh_-A01 [9] completely inhibited Cl^−^ current.

### HPLC fractionation and structure determination

**Figure 2:**
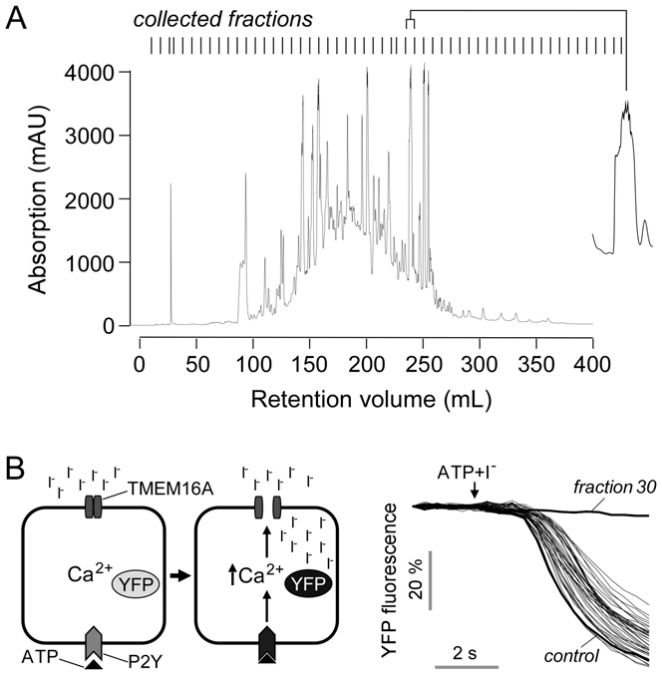
Fractionation of Thai herbal formulation. A. HPLC fractionation showing the chromatogram at 280 nm absorbance. Vertical lines at the top show 53 collected fraction with expanded view of fraction 30 shown at the right. B. (left) Principle of cell-based fluorescence plate-reader assay. TMEM16A-facilitated I^−^ influx was measured from the kinetics of decreasing YFP-H148Q/I152L/F46L fluorescence in response to addition of I^−^ solution containing the P2Y agonist ATP, which elevates cytoplasmic Ca^2+^ level. (right) Inhibition by HPLC fractions shows the majority of activity resides in fraction 30.

Fig. 2A shows HPLC fractionation of the Thai herbal formulation. There were approximately 20 distinct peaks in the chromatogram overlying a broad peak. Each of the 53 collected fractions was tested for inhibition of TMEM16A using a plate-reader assay with FRT cells expressing the I^−^-sensitive fluorescent protein YFP-H148Q/I152L/F46L and human TMEM16A. As diagrammed in Fig. 2B (left) I^−^ addition reduced cellular fluorescence following TMEM16A activation by ATP. Fig. 2B (right) shows that fraction 30 fully inhibited TMEM16A-mediated I^−^ influx, whereas the other fractions had little effect. Fig. 2A shows an expanded view of fraction 30.

**Figure 3:**
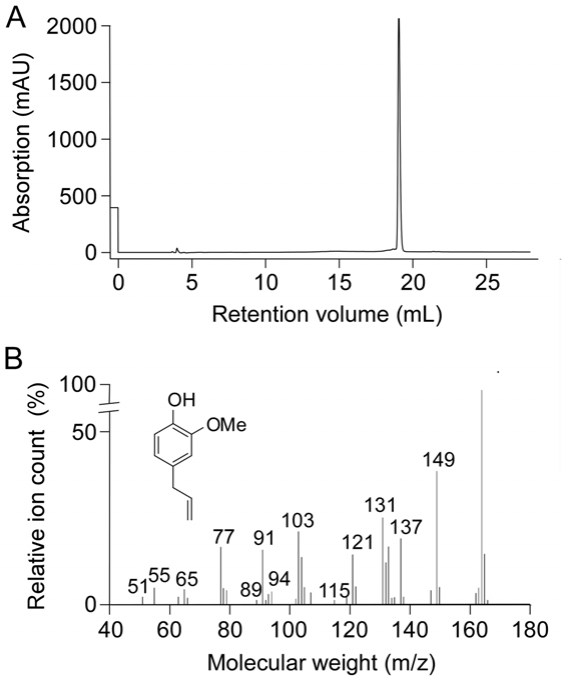
Identification of purified active fraction as eugenol. A. HPLC of fraction 30, showing a single peak. B. High-resolution mass spectra of purified fraction 30. Inset: Structure of eugenol.

Fraction 30 was resolved using a second HPLC column, showing a single peak (Fig. 3A). High-resolution mass spectrometry of the purified material gave the molecular size (164 Da) and known fragmentation pattern of eugenol (Fig. 2B). In order to verify eugenol as compound identity, we obtained ^1^H, ^13^C, COSY, HMQC and HMBC NMR spectra because some structural isomers of eugenol, such as chavibetol, have nearly identical mass spectrometric patterns. NMR spectra of purified fraction 30 were identical to commercial eugenol, as was TMEM16A inhibition potency, confirming compound identity. The concentration of eugenol in the original Thai herbal formulation was ∼200 mM as measured by HPLC against eugenol standards.

### Biological studies

**Figure 4:**
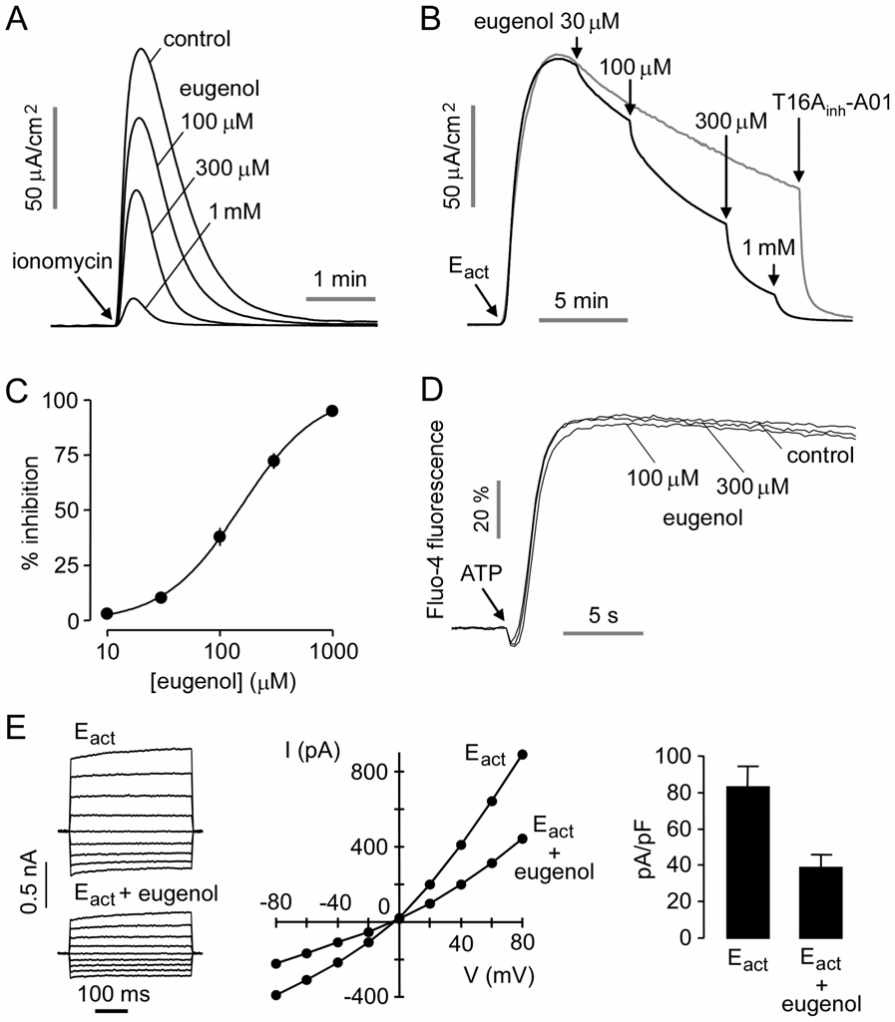
TMEM16A inhibition by eugenol. A. Short-circuit current measured in TMEM16A-expressing FRT cells in the presence of a transepithelial Cl^−^ gradient. Indicated concentrations of eugenol were added 10 min prior to TMEM16A activation by 1 µM ionomycin. B. Short-circuit current measured after TMEM16A activation by 10 µM E_act_, followed by indicated concentrations of eugenol. C. Eugenol concentration inhibition data from measurements in B (mean ± S.E., n=4). D. Eugenol does not inhibit cytoplasmic Ca^2+^ elevation in response to ATP as shown by Fluo-4 fluorescence. Cells were incubated with eugenol for 10 min prior to measurements. E. Whole-cell TMEM16A current recorded at a holding potential of 0 mV, and pulsing to voltages between ± 80 mV (in steps of 20 mV) in the absence and presence of 150 µM eugenol (left). TMEM16A was activated by 5 µM E_act_. Current/voltage (I/V) plot of mean currents at the middle of each voltage pulse (center). Summary of current density data measured at +80 mV (right, mean ± S.E., n=5).

Fig. 4A shows short-circuit current in TMEM16A-expressing FRT cells in which the basolateral membrane was permeabilized with amphotericin B and a transepithelial Cl^−^ gradient was applied. Addition of eugenol 5 min prior to activation of TMEM16A by 1 µM ionomycin produced concentration-dependent inhibition of Cl^−^ current. Fig. 4B showed inhibition of E_act_-stimulated TMEM16A Cl^−^ current by increasing eugenol concentrations. Concentration-inhibition data in Fig. 4C gave a fitted IC_50_ of 154 µM. Eugenol did not inhibit cytoplasmic Ca^2+^ in response to ATP (Fig. 4D). Similar results were obtained for Ca^2+^ elevation following ionomycin treatment (not shown). Whole-cell patch-clamp analysis was done to investigate the engenol inhibition mechanism (Fig. 4E). Application of 150 µM eugenol inhibited TMEM16A chloride current (induced by 5 µM E_act_) at all voltages, indicating a voltage-independent block mechanism.

**Figure 5:**
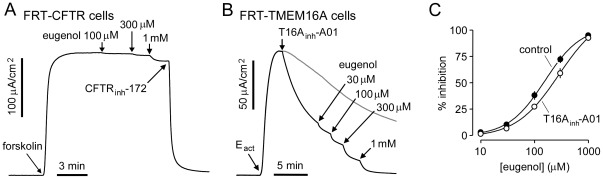
Eugenol selectivity and binding. A. CFTR Cl^−^ current was measured in FRT cells expressing wild type CFTR-expressing in the presence of a transepithelial Cl^−^ gradient and after basolateral membrane permeabilization. CFTR Cl^−^ current was stimulated by forskolin (20 µM), followed by eugenol addition. CFTR Cl^−^ current was completely inhibited by CFTR_inh_-172 (10 µM). B. Investigation of possible synergy/competition of eugenol with T16A_inh_-A01. T16A_inh_-A01 (1 µM) was added to inhibit TMEM16A Cl^−^ current by ∼50% followed by indicated concentrations of eugenol. C. Eugenol concentration inhibition data from measurements in B (mean ± S.E., n=3–4).

The Cl^−^ channel selectivity of eugenol inhibition was studied. Many inhibitors of CaCCs are non-selective Cl^−^ channel inhibitors that also inhibit CFTR [19]. Fig. 5A shows that eugenol, which inhibits TMEM16A by >95% at 1 mM, had little effect on CFTR Cl^−^ conductance. To determine whether the site of action of eugenol on TMEM16A might overlap with that of the small-molecule aminophenylthiazole TMEM16A-selective inhibitor T16A_inh_-A01, we studied whether pre-added T16A_inh_-A01 affected the potency of eugenol (Fig. 5B). Fig. 5C shows that pre-treatment with 1 µM T16A_inh_-A01 increased the eugenol IC_50_ from ∼150 µM to 280 µM, suggesting partial overlap in their binding sites.

**Figure 6:**
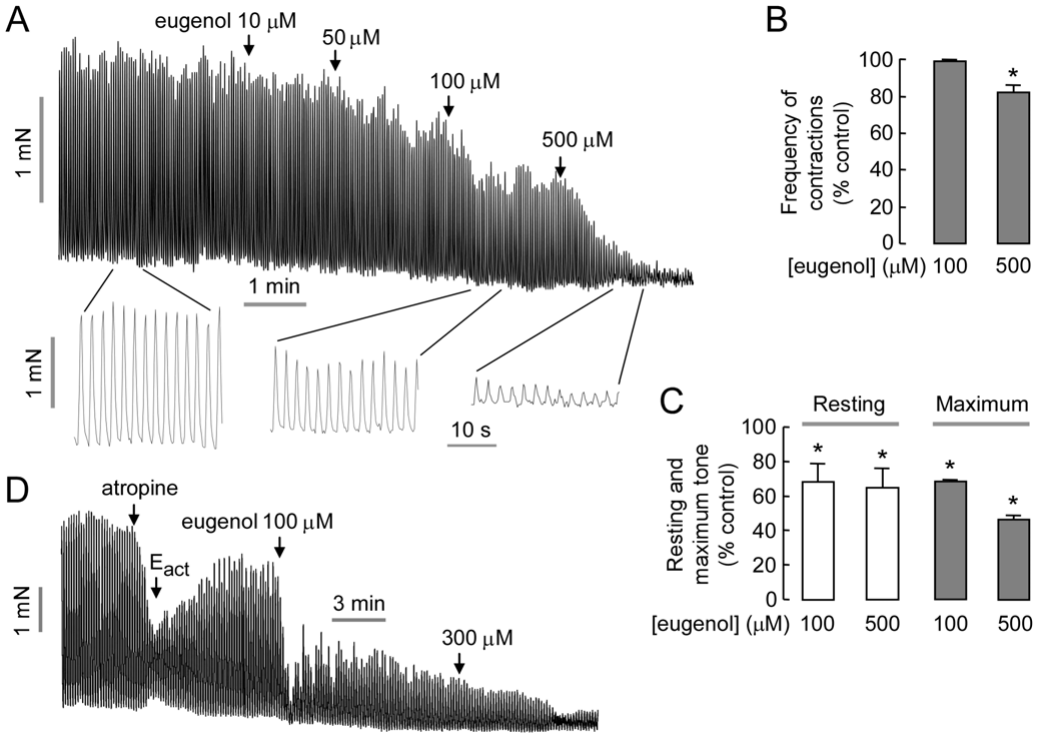
Eugenol inhibits intestinal smooth muscle contraction. A. Representative traces from mouse ileal segments showing eugenol inhibition of contractions. B. Eugenol effect on relative contraction frequency (mean ± S.E., n=4, * P<0.05). C. Eugenol effect on resting and maximum tone (mean ± S.E., n=4, * P<0.05). D. Eugenol inhibition of ileal contraction after stimulation by TMEM16A activator E_act_. Representative of 3 experiments.

TMEM16A is expressed in interstitial cells of Cajal, the pacemaker cells that control smooth muscle contraction in stomach and intestine [8]. We previously showed that inhibition of TMEM16A by T16A_inh_-A01 inhibited mouse intestinal smooth muscle contraction [10]. Eugenol was tested in mouse ileum ex vivo. Fig. 6A shows considerable constitutive activity of mouse ileal muscle segments at baseline, with large, spontaneous intestinal contractions that were inhibited by eugenol in a dose-dependent manner. Eugenol had a small effect on contraction frequency (Fig. 6B), but a more marked effect on resting and maximal tone (Fig. 6C). Fig. 6D shows eugenol inhibition of ileal contractions following activation by E_act_, in which atropine was added initially to reduce basal constitutive activity.

### Structure-activity analysis

Eugenol contains three functional groups - hydroxyl, methoxy and allyl groups - on a single phenyl ring. Table S1 summarizes TMEM16A inhibition activity of eugenol (compound 1) and commercially available analogs (compounds 2–11) at 200 µM. A series of hydroxyl and methoxy combinations at R^1^, R^2^ and R^3^ were tested, as well as different aliphatic chains at R^4^. Inhibition activity required a large hydrophobic group at R^4^. Short hydrocarbon chains (compounds 2, 3 and 6) or a hydrophilic chain (compound 8) reduced inhibition activity. The terminal double bond of allyl group in eugenol is not required the activity (compounds 4, 5 and 7).

## Discussion

Eugenol is an aromatic molecule found in various plants and essential oils. While it is found in basil, cinnamon, Japanese star anise, lemon balm and dill, eugenol is best known as a major component in clove (*Syzygium aromaticum*), making up 85–90% of clove leaf oil, 80–85% of clove bud oil, and 87–92% of clove stem oil [12]. The first biological action of eugenol, antibacterial activity, was reported in 1947 [20]. Subsequently, numerous studies have reported eugenol effects on immune, reproductive, cardiovascular, gastric, nervous and urinary systems [21]. Because of its wide range of activities and its natural abundance, eugenol has become a widely available dietary supplement.

Though eugenol inhibition of Cl^−^ channels has not been reported previously, eugenol has been reported to modulate the activity of several cation channels in neurons, muscle cells and epithelial cells. There is evidence that the well-known analgesic property of eugenol in dentistry, where eugenol is applied topically [22], is related to inhibition of Na^+^ [23], K^+^ [24] and Ca^2+^ [25] channels. A recent report shows that inhibition of TMEM16A in rat sensory neurons block bradykinin-induced nociceptive signals [26]. TMEM16A inhibition by eugenol may thus account, in part, for its analgesic effects when applied topically.

Yeon et al. [27] reported that eugenol inhibited hyperpolarization-activated cyclic nucleotide-gated Na^+^ and K^+^ channels in trigeminal ganglion neurons with IC_50_ of 157 µM. In general, IC_50_ for eugenol activities are 100 µM or greater, which is probably related to its small molecular size. IC_50_ values were ∼300 µM for inhibition of Ca^2+^ current in cardiac myocytes [28], and ∼600 µM for inhibition of tracheal smooth muscle contraction [29]. The concentration of engenol in blood is much lower than typical IC_50_ values, even with high dose oral administration. Lionnet et al. [30] reported eugenol concentrations in rat after oral administration (40 mg/kg) of 0.6 µM in serum and 2.8 µM in brain and spinal cord. A similar study in rats revealed a serum concentration of 1.5 µM [31]. Higher serum concentrations of eugenol of 180 µM were found following intravenous administration [32], suggesting limited oral bioavailability and/or first-pass hepatic metabolism. The glucuronide conjugate of eugenol and eugenol sulfate have been detected in blood and urine [32], [33].

We found here an IC_50_ of ∼150 µM for eugenol for the inhibition of TMEM16A Cl^−^ current in cells and inhibition of intestinal smooth muscle contraction. The Thai herbal formulation contains ∼200 mM eugenol. With a recommended oral dose of the herbal formulation of 5–10 mL and a typical intestine fluid volume of several liters, the intestinal eugenol concentration should be in the range of several hundred micromolar, where it is predicted to inhibit TMEM16A.

In conclusion, we found that eugenol is an inhibitor of TMEM16A Cl^−^ channels, adding to the list of eugenol molecular targets and potentially accounting for its antidiarrheal and analgesic activities.

## Materials and Methods

### Ethics Statement

Protocols were approved by the University of California San Francisco Committee on Animal Research.

### Cell lines and materials

FRT cells stably expressing human TMEM16A and the halide sensor YFP-H148Q/I152L/F46L were generated as described [9]. Cells were plated in 96-well black-walled microplates (Corning Inc., Corning, NY) at a density of 20,000 cells per well in Coon's modified F12 medium supplemented with 5% fetal calf serum, 2 mM L-glutamine, 100 U/mL penicillin and 100 µg/mL streptomycin. T84 cells were cultured in DMEM/Ham's F-12 (1∶1) medium containing 10% FBS, 100 U/ml penicillin and 100 µg/ml streptomycin. A commercially available Thai herbal formulation (Krisanaklan, Osotspa Inc., Bangkapi, Thailand) consists of an ethanol/water (54∶46) extract in which each 100 mL was obtained from 10 g of *Aquilaria crassna* bark, 33.3 g of clove flower bud, 2 g of *Terminalia triptera Stapf* bark and 4.8 g of camphor flower bud. HPLC grade solvents were purchased from Fisher Scientific (Fairlawn, VA) and EMD Chemical Inc. (Philadelphia, PA). Eugenol and eugenol analog 2 were purchased from Sigma-Aldrich (St. Louis, MO). Other eugenol analogs were purchased from Fisher Scientific (Pittsburgh, PA).

### Plate-reader fluorescence assay of I^−^ influx

YFP fluorescence measurements in TMEM16A expressing FRT cells were carried out on a fluorescence plate reader (Optima; BMG Labtech, Durham, NC) equipped with dual syringe pumps and 500±10 nm excitation and 535±15 nm emission filters as described [11]. Each well of a 96-well plate was washed 3 times with PBS (200 µL/wash). The fractions from the Thai herbal formulation (2 µL) and 100 µL PBS were added to each well. After 10 min incubation, each well was assayed individually for TMEM16A-mediated I^−^ influx by recording fluorescence continuously (400 ms per point) for 2 s (baseline), followed by addition of 100 µL of a 140 mM I^−^ solution containing 200 µM ATP. The initial rate of I^−^ influx was computed from fluorescence data by non-linear regression.

### Short-circuit current

Snapwell inserts containing TMEM16A-expressing FRT cells or T84 cells were mounted in Ussing chambers (Physiologic Instruments, San Diego, CA). Activators and inhibitors (herbal formulation, eugenol, ionomycin, tannic acid, forskolin, CFTR_inh_-172, ATP, E_act_, T16A_inh_-A01) were added to the apical solution and an equal volume of vehicle was added at the same time to the basolateral solution. Symmetrical HCO_3_^−^-buffered solutions were used for T84 cells. For FRT cells, the hemichambers were filled with a half-Cl^−^ solution (apical) and the HCO_3_^−^-buffered solution (basolateral), and the basolateral membrane was permeabilized with 250 µg/mL amphotericin B. Cells were bathed for a 10 min stabilization period and aerated with 95% O_2_/5% CO_2_ at 37°C or room temperature. Short-circuit current was measured using an EVC4000 Multi-Channel V/I Clamp (World Precision Instruments, Sarasota, FL).

### Patch-clamp

Whole-cell recordings were made at room temperature on TMEM16A-expressing FRT cells as described [9]. The pipette solution contained (in mM): 130 CsCl, 0.5 EGTA, 1 MgCl_2_, 1 Tris-ATP, and10 HEPES (pH 7.2). The bath solution contained (in mM): 140 NMDG-Cl, 1 CaCl_2_, 1 MgCl_2_, 10 glucose and 10 HEPES (pH 7.4). TMEM16A was activated by 5 µM E_act_ and then inhibited by 150 µM eugenol. Whole-cell currents were elicited by applying hyperpolarizing and depolarizing voltage pulses from a holding potential of 0 mV to potentials between −80 mV and +80 mV in 20 mV steps. Currents were digitized, filtered at 5 kHz, and sampled at 1 kHz.

### Cytoplasmic Ca^2+^ measurement

FRT cells in 96-well plates were loaded with Fluo-4 NW (Invitrogen, Carlsbad, CA) at 48 h after plating. Fluo-4 fluorescence was measured continuously using a FLUOstar Optima fluorescence plate reader (BMG Labtechnologies) equipped with syringe pump for addition of ATP.

### Intestinal smooth muscle contraction

Wild type CD1 mice (age 8–10 weeks) were killed by avertin overdose (200 mg/kg). The ileum was removed and washed with ice-cold HCO_3_^−^-buffered solution. The ends of the ileal segments were tied with silk thread and connected to a force transducer. Ileal segments were equilibrated for 60 min with a resting force of ∼1 mN, with changes of the bathing solution every 15 min. Tension was monitored continuously with a fixed-range precision force transducer (TSD, 125 C; Biopac, Goleta, CA) connected to a differential amplifier (DA 100B; Biopac). Data were recorded using MP100, Biopac digital acquisition system and analyzed using Acknowledge 3.5.7 software.

### High performance liquid chromatography (HLPC)

Fractionation was performed on an AKTAexplore 10 system (GE Healthcare Life Science, Piscataway, NJ) equipped with a C18 reversed-phase column (Varian Pursuit XRs, 250×10 mm, 5 mm particle size, Waldbronn, Germany). After 1 mL of the formulation was injected, the gradient was developed with mobile phase A (0.1% formic acid in water) and mobile phase B (methanol) at 5 mL/min flow rate. The mobile phase was 100% A for 4 min, followed by a linear decrease to 1% A over 40 min, and then 1% A for 40 min. Absorption was recorded at 280 nm. Total eluent was collected in 53 fractions (8 mL/fraction). The purity of the active component, as determined by plate reader assay, was verified on a separate C18 column (Pharmacia Biotech, 250×4.6 mm, 5 mm, Uppsala, Sweden). After 100 µL was injected, the gradient was developed with mobile phase A and mobile phase C (acetonitrile) at 1 mL/min flow rate.

### Structure characterization

NMR spectra were obtained on a Bruker 300 MHz instrument (Madison, WI). HRMS was done at the High Resolution Mass Spectrometry Facility at the University of California, Riverside.

## Supporting Information

Table S1 **Structure-activity analysis of eugenol analogs.** TMEM16A inhibition was measured by fluorescence plate reader assay.
(DOC)
