# Supplementary material for: Fractionation of a Herbal Antidiarrheal Medicine Reveals Eugenol as an Inhibitor of Ca2+-Activated Cl− Channel TMEM16A
Source: PLoS One. 2012 May 30;7(5):e38030. doi: 10.1371/journal.pone.0038030 (PMC3364195; doi:10.1371/journal.pone.0038030)
Supplement: Table S1 — Structure-activity analysis of eugenol analogs. TMEM16A inhibition was measured by fluorescence plate reader assay. (DOC) [file pone.0038030.s001.doc]

**Table S1.** Structure-activity analysis of eugenol analogs.

|  | R^1^ | R^2^ | R^3^ | R^4^ | % inhibition at 200 μM |
| --- | --- | --- | --- | --- | --- |
| 1 |  |  |  |  | 51 ± 7 |
| 2 |  |  |  |  | 11 ± 4 |
| 3 |  |  |  |  | 6 ± 5 |
| 4 |  |  |  |  | 39 ± 3 |
| 5 |  |  |  |  | 38 ± 7 |
| 6 |  |  |  |  | 35 ± 7 |
| 7 |  |  |  |  | 74 ± 5 |
| 8 |  |  |  |  | 9 ± 4 |
| 9 |  |  |  |  | 19 ± 5 |
| 10 |  |  |  |  | 68 ± 4 |
| 11 |  |  |  |  | 47 ± 8 |
